# Selective killing of *K-ras*–transformed pancreatic cancer cells by targeting NAD(P)H oxidase

**DOI:** 10.1186/s40880-015-0012-z

**Published:** 2015-04-08

**Authors:** Peng Wang, Yi-Chen Sun, Wen-Hua Lu, Peng Huang, Yumin Hu

**Affiliations:** Sun Yat-sen University Cancer Center; State Key Laboratory of Oncology in South China; Collaborative Innovation Center for Cancer Medicine, Guangzhou, Guangdong 510060 P.R. China; Institute of Cardiopulmonary Cerebral Resuscitation, Sun Yat-sen Memorial Hospital, Sun Yat-sen University, Guangzhou, Guangdong 510000 P.R. China; Department of Translational Molecular Pathology, The University of Texas MD Anderson Cancer Center, Houston, TX 77030 USA

**Keywords:** K-ras, Pancreatic cancer, Reactive oxygen species, NADPH oxidase, Capsaicin

## Abstract

**Introduction:**

Oncogenic activation of the *K*-*ras* gene occurs in >90% of pancreatic ductal carcinoma and plays a critical role in the pathogenesis of this malignancy. Increase of reactive oxygen species (ROS) has also been observed in a wide spectrum of cancers. This study aimed to investigate the mechanistic association between *K-ras*–induced transformation and increased ROS stress and its therapeutic implications in pancreatic cancer.

**Methods:**

ROS level, NADPH oxidase (NOX) activity and expression, and cell invasion were examined in human pancreatic duct epithelial E6E7 cells transfected with *K*-*ras*^G12V^ compared with parental E6E7 cells. The cytotoxic effect and antitumor effect of capsaicin, a NOX inhibitor, were also tested *in vitro* and *in vivo*.

**Results:**

*K-ras* transfection caused activation of the membrane-associated redox enzyme NOX and elevated ROS generation through the phosphatidylinositol 3′-kinase (PI3K) pathway. Importantly, capsaicin preferentially inhibited the enzyme activity of NOX and induced severe ROS accumulation in *K-ras*–transformed cells compared with parental E6E7 cells. Furthermore, capsaicin effectively inhibited cell proliferation, prevented invasiveness of *K-ras*–transformed pancreatic cancer cells, and caused minimum toxicity to parental E6E7 cells. *In vivo*, capsaicin exhibited antitumor activity against pancreatic cancer and showed oxidative damage to the xenograft tumor cells.

**Conclusions:**

K-ras oncogenic signaling causes increased ROS stress through NOX, and abnormal ROS stress can selectively kill tumor cells by using NOX inhibitors. Our study provides a basis for developing a novel therapeutic strategy to effectively kill *K-ras*–transformed cells through a redox-mediated mechanism.

## Background

Oncogenic mutations of the *KRAS* gene are present in >90% of pancreatic ductal carcinoma [1], which is an aggressive and deadly cancer [2]. Since pancreatic ductal carcinoma is unusually resistant to chemotherapy and radiation therapy and little progress has been achieved in the treatment of pancreatic cancer, surgical resection remains to be the only potentially curative therapy. The potential discoveries of pancreatic cancer therapeutics rely on advances in our understanding of the biology of the disease. Genetic lesions, including mutations of V-Ki-ras2 Kirsten rat sarcoma viral oncogene homolog (*KRAS*), cyclin-dependent kinase inhibitor 2A (*CDKN2A*), tumor protein 53 (*TP53*), breast cancer 2 (*BRCA2*), and mothers against decapentaplegic homolog 4 (*SMD4/DPC4*), have been thought to contribute to the evolution of pancreatic adenocarcinoma [3]. Activating *KRAS* mutations are found in more than 90% of pancreatic adenocarcinomas and are highly associated with disease progression due to the activation of several effector pathways that induce cell proliferation, survival, invasion, and metabolic alterations [3-5]. Given the almost ubiquitous occurrence of *K*-*ras* mutations and its critical role in the development of pancreatic cancer, the ideal therapeutic strategy would be the direct blocking of KRAS oncogenic signaling. However, an effective small-molecule inhibitor of KRAS has yet to be identified [6].

Whereas the major effector proteins, such as Raf kinase, phosphatidylinositol 3′-kinase (PI3K), and RalGDS, play vital roles in Ras transformation, accumulating evidence has shown that reactive oxygen species (ROS) may serve as a messenger of Ras in signaling transduction pathways and that moderate increases in ROS levels may promote cell proliferation and contribute to cancer development [7,8]. Therefore, ROS appear to be an important downstream effector of Ras transformation in cancer cells. The role of the membrane-associated NADPH oxidase (NOX) in non-mitochondrial formation of ROS has been observed in various studies [9-11]. The activation or up-regulation of NOX has also been shown to play an important role in maintaining the cancer phenotype through stimulating the production of ROS [12-14]. The previous findings prompted us to investigate whether K-ras oncogenic signaling increases ROS levels through the activation of NOX and whether modulators of NOX could provide a potential therapeutic opportunity for pancreatic cancer through a redox-mediated mechanism. Capsaicin (8-methyl-*N*-vanillyl-6-nonenamide), a natural compound, is a pungent ingredient found in a variety of red peppers and has been shown to inhibit cell surface NOX activity [15,16]. In the current study, we aimed to determine the mechanistic role of NOX in mediating ROS generation induced by K-ras oncogenic signaling. We compared ROS production as well as the expressions and activities of NOX in parental human pancreatic duct epithelial E6E7 cells and *K-ras*–transformed E6E7 cells, which have previously been shown to be highly tumorigenic [17]. We also examined the effect of capsaicin on parental and *K-ras*–transformed E6E7 cells. Importantly, the role of NOX-derived ROS generation in capsaicin-induced cytotoxicity was tested in *K-ras*–transformed E6E7 cells in comparison with parental E6E7 cells.

## Methods

### Antibodies and reagents

The following antibodies were used for immunoblotting analysis using standard Western blotting procedures: superoxide dismutase 1 (SOD1), superoxide dismutase 2 (SOD2), p22phox, and p-p40phox were purchased from Santa Cruz Biotechnology, Dallas, TX, USA; β-actin, tublin, diphenyleneiodonium chloride (DPI), and capsaicin were purchased from Sigma-Aldrich, St. Louis, MS, USA.

### Cell culture

The parental E6E7 cell line and *K-ras*–transformed cell line, which had been established by transfecting the immortalized human pancreatic duct epithelial E6E7 cell line with *K-ras*^G12V^, were kindly provided by Dr. Paul Chiao from The University of Texas, MD Anderson Cancer Center and were cultured as reported previously [17]. Primary pancreatic cancer cell lines, including AsPC-1, Capan-1, and Panc-1, were obtained from American Type Culture Collection (ATCC) and cultured in Dulbecco’s Modified Eagle’s medium (DMEM) with 10% Fetal bovine serum (FBS).

### Quantitative real-time Polymerase Chain Reaction (PCR) analysis

The sequences for the genes to be measured are as follows: 5′-GGAGTTTCAAGATGCGTGGAAACTA-3′ (sense) and 5′-GCCAGACTCAGAGTTGGAGATGCT-3′ (antisense) for *NOX2*, 5′-CAAGCCGTGACCAAGGACACCTG-3′ (sense) and 5′-CACACAGGACATCCACCGTGTC-3′ (antisense) for *NOXA1*. Real-time PCR analysis was performed by using the SYBR Premix Ex Taq II kit (TaKaRa Bio, Otsu, Shiga, Japan) and Real-Time PCR Detection Systems (Bio-Rad, Hercules, CA, USA).

### MTT assay

Cell growth was determined using MTT reagent in 96-well plates. After incubation, 20 μL MTT reagent was added to each well and incubated for an additional 4 hours and then the supernatant was removed. The cell pellets were dissolved in 200 μL DMSO. Absorbance was determined using a MultiSkan plate reader (Thermo, Helsinki, USA) at a wavelength of 570 nm.

### Colony formation assay

Cells were seeded in six-well plates and cultured for about 2 weeks. Colonies were fixed with methanol for 10 minutes and stained with crystal violet solution (Beyotime, Jiangsu, China) for 30 minutes. All the experiment was repeated 3 times.

### NOX activity

Cells were suspended in lysis buffer containing 20 mmol/L HEPES, 10 mmol/L KCl, 1.5 mmol/L MgCl_2_, 1 mmol/L EDTA,1 mmol/L EGTA, 100 mmol/L sucrose, and a cocktail of protease inhibitors. After homogenization, the samples were centrifuged at 800 *g* at 4°C for 5 minutes to pellet unbroken cells and nuclei. The supernatants were centrifuged at 100,000 *g* for 30 minutes to separate the membrane fraction (pellet) and the cytosolic fraction (supernatant). NOX activity was measured by lucigenin-derived chemiluminescence, with 100 μmol/L NADPH or NADH as substrate, 50 μmol/L lucigenin, and 25 μg of cell membrane proteins. Chemiluminescence was measured using a luminometer (Turner Designs, Sunnyvale, CA, USA) for 1 minute. The signal was normalized and expressed as arbitrary light units per microgram protein per minute.

### Rac activity

The Rac activity assay was performed using the Rac-GEF (guanine-nucleotide exchange factors) Assay Kit (Cell Biolabs, San Diego, CA, USA). Briefly, cells were washed in cold PBS, lysed in 1× Assay/Lysis Buffer, and centrifuged for 10 minutes at 14,000 *g* at 4°C. Aliquots from the supernatant were used for determining protein concentration. The supernatant was incubated with nucleotide-free Rac1 G15A agarose beads to pull down the active form of Rac-GEFs. The beads were washed 3 times with 1× Assay/Lysis Buffer, and the bound proteins were eluted. The active Rac proteins were detected by Western blotting using an anti-Rac-GEF antibody (Tiam1).

### Invasion assay

Invasion assays were performed with BD BioCoat Matrigel Invasion Chambers (BD Biosciences, San Jose, CA, USA). Pre-coated filter Matrigel inserts were re-hydrated with 0.5 mL of PBS for 2 hours in humidified tissue culture incubator at 37°C in 5% CO_2_ atmosphere. After rehydration, PBS was removed. Then, 1 × 10^5^ parental or *K-ras*–transformed E6E7 cells and Capan-1, AsPC-1, Panc-1 cells in 0.5 mL of supplement-free medium with or without 10 μmol/L capsaicin were seeded onto the upper part of each chamber insert, and the 24-well plates were filled with 0.5 mL of their culture medium. Following incubation for 16 hours, non-invaded cells on the upper surface of the insert were wiped off with a cotton swab, and the cells that had migrated onto the lower surface of the filter, were fixed and stained with the Hema 3 Manual Staining System (Fisher Scientific, Pittsburgh, PA, USA) containing a fixative and 2 stain solutions. The inserts were air dried and photographed. Invasiveness was determined by counting cells in 3 microscopic fields (×100) per well, and the extent of invasion was expressed as an average number of cells per microscopic field.

### Measurement of ROS production and ATP generation

Cells were stained with 100 ng/mL hydroethidine (HET) (Invitrogen, Carlsbad, CA, USA) and 5 μmol/L DCF-DA (Invitrogen, Carlsbad, CA, USA) for 60 minutes before the measurement of superoxide and hydrogen peroxide using a FACScan flow cytometer (Becton Dickinson, Franklin Lakes, NJ, USA). Cellular ATP generation was measured using CellTiter-Glo Luminescent Cell Viability Assay kit (Promega, Wisconsin, USA) according to manufacturer’s recommendations.

### Animal study

Four-week-old, BALB/c male nude mice were purchased from Medical Experimental Animal Center of Guangdong Province, China. A total of 2 × 10^6^ AsPC-1 cells were inoculated into the right flanks of the mice by subcutaneous injection. When the volume of tumors reached 100 mm^3^, the mice were randomly divided into 2 groups of 10 mice each. The treatment group received 15 mg/kg capsaicin in 0.9% sodium chloride solution (intraperitoneal injection, 3 times per week). The control group received equal volume of 0.9% sodium chloride solution by intraperitoneal injection. Five weeks after inoculation, all mice were euthanized and the tumor weights were measured. Animal experiments were approved by Institutional Animal Care and Use Committee of Sun Yat-sen University Cancer Center and performed under the guidelines of the Care and Use of Laboratory Animals (NIH publications Nos. 80–23, revised 1996).

### Immunohistochemistry and TUNEL assay

Representative tumor tissues were sectioned and embedded in paraffin. The slides were then incubated with the primary antibody (mouse anti–8-oxoguanine monoclonal antibody, Abcam, Cambridge, UK) at 1:200 dilution overnight in a humidified chamber at 4°C. The slides were washed and incubated with horseradish peroxidase-conjugated secondary antibody (Envision Detection Kit, Dako, Glostrup, Denmark) at 37°C for 30 minutes. Finally, the samples were stained with 3, 3-diaminobenzidine (DAB) solution and counterstained with hematoxylin and eosin (HE). Tumor cell death induced by capsaicin was detected by TUNEL assay with the In Situ Cell Death Detection Kit (Roche, Indianapolis, IN, USA) according to manufacturer’s instructions.

### Statistical analysis

Statistical significant differences were evaluated by using Student’s *t* test (Prism GraphPad, San Diego, CA, USA). The Kolmogorov-Smirnov test (Cell Quest Pro software, Becton-Dickinson, San Jose, CA, USA) was used to evaluate the significant difference between control and treatment groups in flow cytometry analysis. A *P* value of <0.05 was considered statistically significant.

## Results

### Oncogenic transformation induced by *K-ras* increased ROS generation

**Figure 1:**
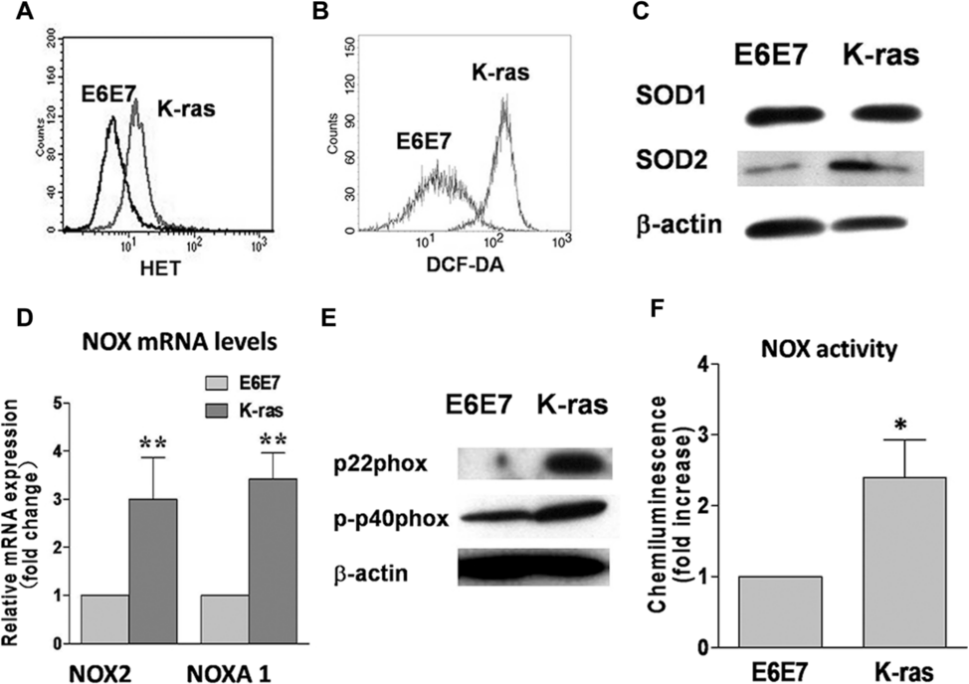
**Increase in oxidative stress and activation of NOX in** ***K-ras*** **–transformed pancreatic cancer cells. A**, comparison of basal superoxide levels in parental (E6E7) and *K-ras*–transformed E6E7 cells (K-ras). *K-ras*–transformed cells exhibit a 2-fold increase in superoxide generation detected by fluorescence probe HET. **B**, comparison of basal hydrogen peroxide levels in parental and *K-ras*–transformed E6E7 cells. *K-ras*–transformed cells exhibit a 3-fold increase in hydrogen peroxide generation detected by fluorescence probe DCF-DA. **C**, the levels of CuZnSOD (SOD1) and MnSOD (SOD2) were both up-regulated, as assessed by Western blotting analysis. **D**, total mRNA levels of NOX2 and NOXA1 measured by real-time polymerase chain reaction (PCR). **E**, the levels of p22phox and phosphorylated p40phox were both up-regulated, as assessed by Western blotting analysis. **F**, NOX activity was measured in the presence of 100 μmol/L NADPH or NADH by lucigenin-derived chemiluminescence in 50 μg of membrane fraction from parental and *K-ras*–transformed E6E7 cells. The values are shown as the mean ± standard deviation (SD). *, *P* < 0.05; **, *P* < 0.01. NOX, NADPH oxidase; HET, hydroethidine.

To test the hypothesis that *K-ras* transformation activates NOX and renders the transformed cells vulnerable to NOX inhibition through further ROS stress, we first evaluated the effect of oncogenic *K-ras* on ROS production. As shown in Figure 1A and B, *K-ras*–transformed cells exhibited 2-fold and 5-fold increases in superoxide (O_2_^−^) and hydrogen peroxide (H_2_O_2_) production compared with parental E6E7 cells. The levels of both SOD1 and SOD2, 2 major forms of SOD, were significantly up-regulated in *K-ras*–transformed cells, indicating the increased levels of cellular ROS stress in the transformed cells (Figure 1C).

As NOX has been reported to be a multi-subunit redox enzyme that generates non-mitochondrial source of ROS [10,18], in the current study, we examined the effect of *K-ras* transformation on NOX expression and enzyme activity. The mRNA levels of 2 members of the NOX family, NOX2 and NOXA1, were up-regulated by more than 3-fold in *K-ras*–transformed cells (Figure 1D). The activation of the NOX complex requires proper assembly of the plasma membrane-binding and cytosolic protein components, and the activation is initiated by phosphorylation of the cytosolic complex [19,20]. Western blotting analysis showed that p22phox, the major membrane-binding component, was markedly increased in the transformed cells. Importantly, the level of p40phox, the phosphorylated form of the cytosolic subunit, was also up-regulated in the transformed cells (Figure 1E). The *K-ras*–transformed cells also exhibited a more than 2-fold increase in the enzyme activity of NOX (Figure 1F) compared with parental E6E7 cells, indicating that NOX was indeed activated in the transformed cells.

### Capsaicin preferentially induced ROS production and selectively inhibited NOX activity in *K-ras*–transformed cells

**Figure 2:**
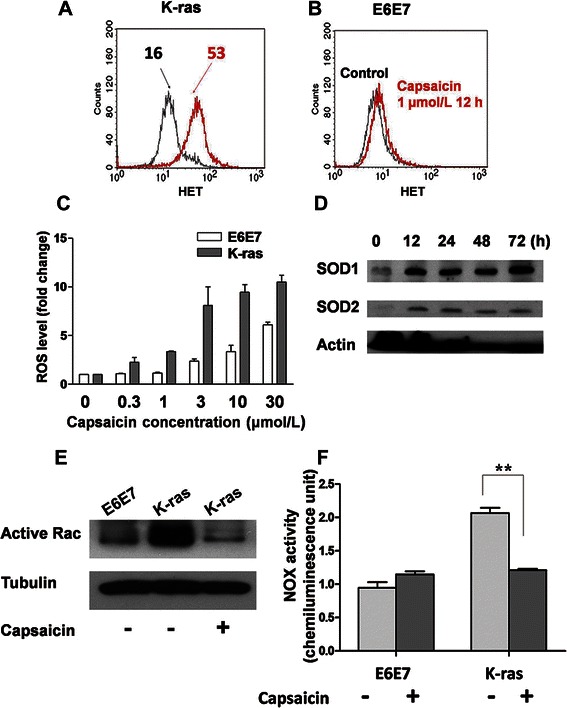
**Capsaicin caused preferential reactive oxygen species (ROS) accumulation and selectively inhibited NOX activity in** ***K-ras*** **–transformed E6E7 cells. A**, incubation with 1 μmol/L capsaicin for 12 hours caused tremendous ROS accumulation in *K-ras*–transformed cells as compared with untreated *K-ras*–transformed cells (superoxide mean level, 16 vs. 53). **B**, the same treatment caused a minimum effect on ROS production in E6E7 cells as compared with untreated parental E6E7 cells (superoxide mean level, 8 vs. 9). **C**, the dose-dependent increase in superoxide generation induced by capsaicin in parental and *K-ras*–transformed E6E7 cells. **D**, treatment with 10 μmol/L capsaicin caused a significant increase in both SOD1 and SOD2 in *K-ras*–transformed cells. **E**, total cell lysates of untreated parental E6E6 cells, untreated *K-ras*–transformed E6E6 cells, and *K-ras*–transformed E6E6 cells treated with 10 μmol/L capsaicin for 12 hours were pulled down with nucleotide-free Rac1 G15A agarose beads. The precipitated active Rac was detected by anti–Rac-GEF antibody. **F**, E6E7 and *K-ras*–transformed cells were pretreated with 10 μmol/L capsaicin for 12 hours. NOX activity was measured in the presence of 100 μmol/L NADPH by lucigenin-derived chemiluminescence in the plasma membrane fraction of parental and *K-ras*–transformed E6E7 cells, with or without capsaicin treatment. The values are shown as the mean ± SD. **, *P* < 0.01.

The significant difference between parental and *K-ras*–transformed cells in NOX expression and enzyme activity prompted us to compare the effects of a NOX inhibitor in these 2 cell lines. Capsaicin has been reported to target NOX and induce cell death in various cancers, including hepatoma and leukemia [16,21]. In the current study, the cellular response to capsaicin revealed a striking selective activity against *K-ras*–transformed cells, compared with parental E6E7 cells. As shown in Figure 2A, 1 μmol/L capsaicin induced an approximately 3-fold increase in ROS production (superoxide) in *K-ras*–transformed cells after 12 hours of treatment. In contrast, treatment with the same concentration of capsaicin did not cause any detectable increase in ROS in parental E6E7 cells (Figure 2B). Quantitative analysis of various concentrations of capsaicin treatment further demonstrated the selective induction of ROS generation in *K-ras*–transformed cells (Figure 2C). Western blotting analysis showed that 12 to 72 hours after the treatment with 10 μmol/L capsaicin, the expression of both SOD1 and SOD2 was significantly increased in *K-ras*–transformed cells, indicating the sustained ROS stress induced by capsaicin (Figure 2D).

To determine the biochemical basis for the selective effect of capsaicin on the context of ROS generation in *K-ras*–transformed cells, NOX activity was assessed before and after capsaicin treatment. NOX is a multi-subunit enzyme, and the regulatory components, p40phox, p47phox, and p67phox, as well as the small GTPase, Rac, are localized in the cytosol during the resting state [9]. Upon activation, GDP is exchanged for GTP, and Rac dissociates from the cytosolic complex, leading to the activation of NOX activity [9]. Western blotting analysis showed that the level of functionally active form of Rac was up-regulated in *K-ras*–transformed cells. Importantly, the activation of Rac was significantly inhibited after 12 hours of treatment with10 μmol/L capsaicin (Figure 2E). In accordance with the inhibition of active Rac by capsaicin, NOX activity in *K-ras*–transformed cells was also suppressed by approximately 50% when they were treated with 10 μmol/L capsaicin for 12 hours (Figure 2F).

### Capsaicin induced selective cytotoxicity in *K-ras*–transformed cells

**Figure 3:**
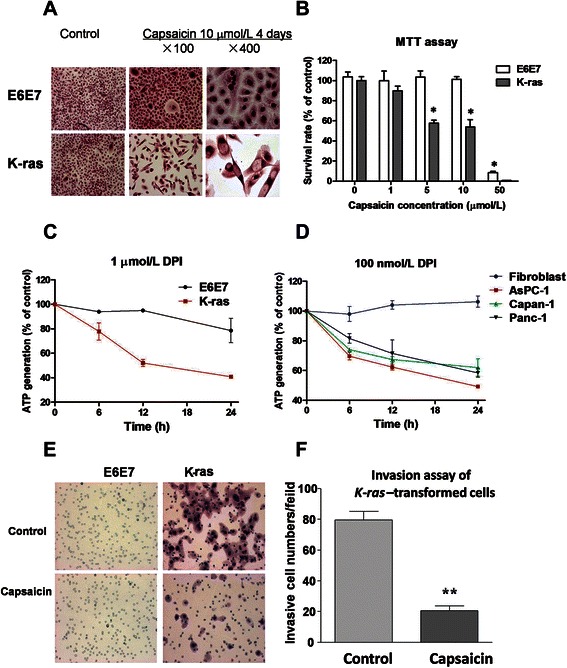
**Selective killing and invasion inhibition of** ***K-ras*** **–transformed cells by capsaicin. A**, parental and *K-ras*–transformed E6E7 cells were incubated with or without 10 μmol/L capsaicin for 4 days in chamber slides. The cells were then stained with hematoxylin and eosin, which show the selective cytotoxicity of capsaicin to *K-ras*–transformed pancreatic cancer cells (magnification, ×100 and × 400). **B**, inhibition of cell proliferation by capsaicin in parental and *K-ras*–transformed E6E7 cells. Cell growth inhibition was measured by long-term MTT assay (mean ± SD of 3 experiments; *P < 0.05). **C**, measurement of ATP generation after treatment of 1 μmol/L diphenyleneiodonium (DPI) in parental and *K-ras* **–**transformed E6E7 cells at various time points as indicated (mean ± SD of 3 experiments). **D**, measurement results of ATP generation after treatment of 100 nmol/L DPI in normal fibroblasts and naturally occurring pancreatic cancer cells at various time points as indicated (mean ± SD of 3 experiments). **E**, parental and *K-ras*–transformed E6E7 cells were seeded onto the chamber insert with a layer of Matrigel matrix, with or without 10 μmol/L capsaicin. Then, 24 hour later, the cells that had digested the Matrigel and migrated to the lower surface of the insert were stained and photographed (magnification, ×100). **F**, the invasion of *K-ras*–transformed cells was assessed by counting the number of cells that had migrated onto the lower surface of the insert. The results are presented as the mean ± SD of the numbers from 3 microscopic fields (magnification, ×100). **, *P* < 0.01.

The significant difference between parental and *K-ras*–transformed E6E7 cells in response to capsaicin-induced ROS accumulation prompted us to compare the cytotoxic effect of this compound in these 2 cell lines. HE staining was used to compare the morphology of these cells before and after treatment. As shown in Figure 3A, 4 days of treatment with 10 μmol/L capsaicin showed minimal effects on parental E6E7 cells. In sharp contrast, after identical treatment, the cell numbers decreased and drastic morphological alterations presented in *K-ras*–transformed cells as compared with the untreated control cells. Because parental E6E7 cells could not form colonies, an adapted MTT assay was used to compare the effect of capsaicin on long-term cell proliferation in parental and *K-ras*–transformed E6E7 cells. As shown in Figure 3B, capsaicin exhibited greater inhibition on *K-ras*–transformed cells after a 14-day period of incubation. To further test the hypothesis that *K-ras* transformation activates NOX and renders the transformed cells vulnerable to NOX inhibitor, DPI, a potent and specific inhibitor of flavoproteins including NAD(P)H oxidase [22], in pancreatic cancer cells and parental E6E7 cells was compared. As shown in Figure 3C and D, ATP generation levels in *K-ras*–transformed E6E7 cells and other pancreatic cancer cells were substantially decreased in a time-dependent manner after treatment with 1 μmol/L and 100 nmol/L DPI, and reached approximately 60% inhibition 24 hours after the treatment. In contrast, the same treatment showed no detectable cytotoxic effect on fibroblasts and only 20% inhibition on parental E6E7 cells.

Because pancreatic cancer is also known to be an aggressive cancer with enhanced invasiveness, the effects of capsaicin on cell migration and invasion were also tested in parental and *K-ras*–transformed E6E7 cells. The invasion measurement was performed with BD chamber inserts coated with a layer of Matrigel matrix. The invasive ability was measured by counting the number of cells that digested the Matrigel and passed through the filter membrane. Consistently, the number of *K-ras*–transformed cells with invasive ability was larger than that of parentalE6E7 cells (Figure 3E). After 24 hours of treatment with 10 μmol/L capsaicin, the invasive ability of K-*ras*–transformed E6E7 cells was significantly inhibited (Figure 3E). The treatment of capsaicin for 24 h inhibited the invasion of *K-ras*–transformed cells by more than 70% (*P* < 0.01) (Figure 3F). Taken together, capsaicin not only showed selective cytotoxicity but also inhibited invasion in malignant cells, either *K-ras*–transformed pancreatic cancer cells or primary pancreatic cancer cells, whereas both effects were not shown in parental E6E7 cells.

### Capsaicin effectively suppressed the proliferation of pancreatic cancer cells and exhibited antitumor activity *in vivo*

**Figure 4:**
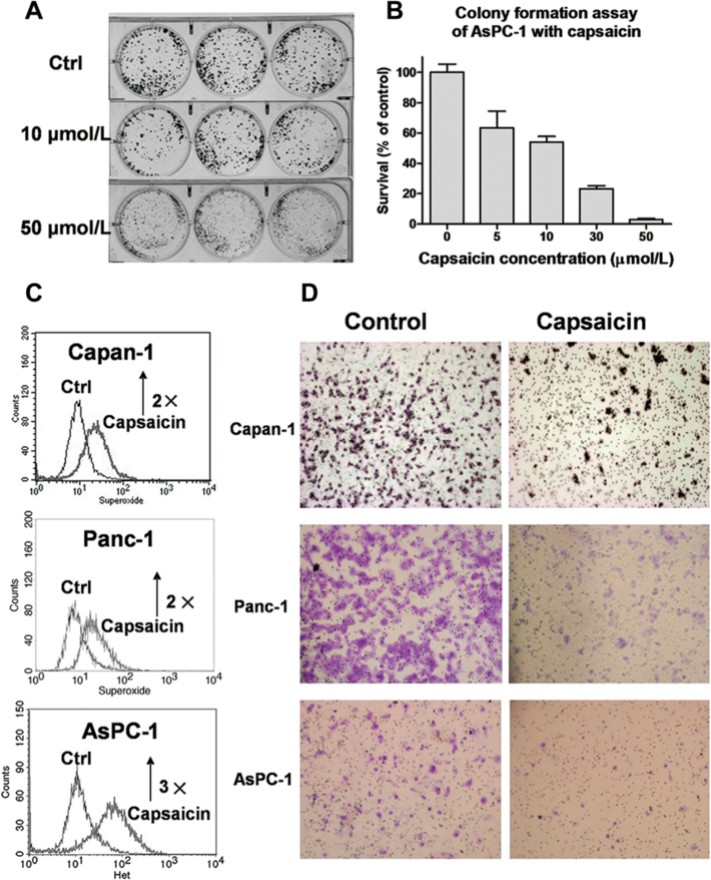
***In vitro*** **antitumor activity of capsaicin against the pancreatic cancer cell lines. A**, equal numbers of AsPC-1 cells were seeded in 6-well plates in triplicates and incubated with various concentrations of capsaicin (10 and 50 μmol/L) for 14 days. The cells were then fixed, stained, and photographed. **B**, quantitative analysis of cytotoxic activity of capsaicin in AsPC-1 cells analyzed by colony formation assay. The data are shown as the mean ± SD of 3 experiments. **C**, the treatment with 30 μmol/L capsaicin for 24 hours induced a 2- to 3-fold increase of cellular superoxide in naturally occurring pancreatic cancer Capan-1, Panc-1, and AsPC-1 cells. **D**, effect of capsaicin on the invasion of naturally occurring pancreatic cancer cells analyzed by BD BioCoat Chamber as described in the Methods section. Cells were seeded onto the chamber insert with or without 30 μmol/L capsaicin treatment. After 24 hours of treatment, cells that had migrated to the lower surface of the insert were subjected to staining and photographed (magnification, ×400). Ctrl, control.

The selective cytotoxicity induced by capsaicin in *K-ras*–transformed cells prompted us to further test its effect in cancer cell lines. The pancreatic cancer cell line AsPC-1 is known to harbor a *K-ras* mutation at codon 12 [23]. As shown in Figure 4A, capsaicin was effective in inhibiting the proliferation of AsPC-1 cells in a colony formation assay, with the half maximal inhibitory concentration (IC_50_) value of approximately 10 μmol/L. Approximately 75% of the proliferation capacity of AsPC-1 cells was inhibited when treated with 30 μmol/L capsaicin (Figure 4B). Interestingly, after 24 hours of treatment with 30 μmol/L capsaicin, a significant accumulation of superoxide was shown in the pancreatic cancer AsPC-1, Panc-1, and Capan-1 cells (Figure 4C). The invasion assay carried out with Capan-1, Panc-1, and AsPC-1 cells revealed that the motility of these pancreatic cancer cells was also substantially inhibited by pretreatment with 30 μmol/L capsaicin for 24 hours (Figure 4D).

**Figure 5:**
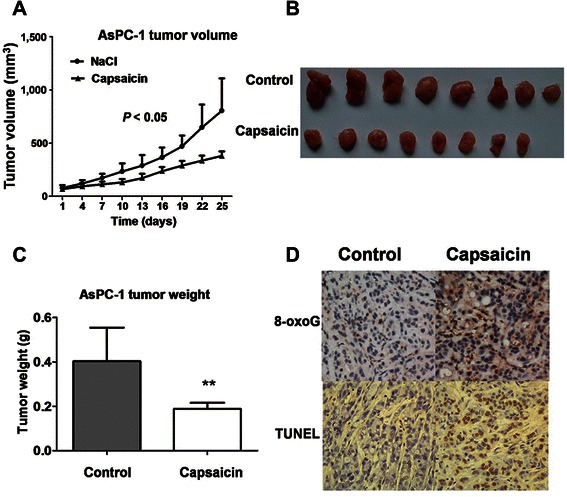
***In vivo*** **antitumor activity of capsaicin against pancreatic cancer. A**, AsPC-1 cells were subcutaneously inoculated into the right flanks of the mice, and the mice were divided into 2 groups, as described in the Methods section. The tumor sizes of the treatment and control groups were measured and compared at the indicated time points and are presented as the mean volume ± SD (*P* < 0.05). **B**, comparison of the size of excised tumors from the treatment and control groups. **C**, the weights of the tumors from the treatment and control groups were measured. Error bars indicate SD (*P* < 0.01, *n* = 10). **D**, tissue slices of AsPC-1 tumors with or without capsaicin treatment were subjected to TUNEL assay and staining for 8-oxoguanine (8-oxoG) (magnification, ×200).

We further detected the activity of capsaicin against pancreatic cancer growth *in vivo*. Capsaicin significantly impaired the tumor growth of AsPC-1 cell xenografts in nude mice, as evidenced by a decrease in tumor volume (*P* < 0.05) and tumor weight in the treatment group (Figure 5A and B). However, the treatment of 15 mg/kg capsaicin did not cause cany toxic effects on the mice, as the mouse weights were not affected by the treatment (data not shown). Twenty-five days after cell inoculation, the average tumor weight in the control group was 403 mg, whereas that in the treatment group was 188 mg (*P* < 0.01) (Figure 5C). The tumor sections were subjected to TUNEL assay and immunostaining for 8-oxoguanine to detect DNA fragmentation and DNA lesions resulting from ROS, respectively [24]. The positive staining of TUNEL and 8-oxoguanine in the treatment group revealed that oxidative damage was induced by capsaicin in AsPC-1cells (Figure 5D). No severe oxidative damage was observed in the control group. Together, these data suggest that capsaicin inhibited pancreatic cancer growth likely through a redox-mediated mechanism.

## Discussion

The association between *K-ras* mutation and pancreatic cancer has been known for decades. An increase in oxidative stress in cancer cells and its association with cancer progression has also long been recognized. However, the mechanistic link between *K-ras* transformation and intrinsic oxidative stress in pancreatic cancer and its therapeutic implications remain to be investigated.

Using isogenic cell lines, we showed here that compared with the parental human pancreatic duct epithelial E6E7 cells, *K-ras*–transformed pancreatic cancer cells exhibited a substantial increase in basal ROS levels, including both O_2_^−^ and H_2_O_2_, indicating that the primary elevated generation of O_2_^−^ may lead to an increase in H_2_O_2_ due to intracellular conversion. Accordingly, we found a significant up-regulation of both SOD1 and SOD2 in the *K-ras*–transformed cells compared with parental E6E7 cells.

**Figure 6:**
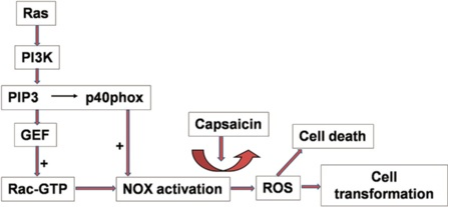
**Proposed model of the selective killing of** ***K-ras*** **–transformed cells by NOX inhibition**. *K-ras* transformation causes the activation of the downstream effector, phosphatidylinositol 3′-kinase (PI3K). Phosphatidylinositol (3,4,5)-trisphosphate (PIP3), the lipid product of a PI3K-catalyzed reaction, enhances the guanine-nucleotide exchange factor (GEF) activity that mediates the exchange of Rac-GDP for Rac-GTP, and provides the lipid necessary for NOX subunit (p40phox) binding upon the translocation to the membrane, therefore causing the activation of NOX. NOX-induced ROS production stimulates cell proliferation and contributes to tumor progression. NOX inhibitor targets the activated NOX and causes excessive ROS generation, leading to cancer cell death. +, activation by the upstream effectors.

It is noteworthy that NOX, the membrane-binding ROS-generating enzyme, has also been suggested to be involved in cell transformation [25,26]. It is known that the activation of NOX requires the proper assembly of multiple regulatory components, including p22phox, ph47phox, p40phox, and p67phox, and the small GTPase, Rac [10,19]. In the current study, NOX expression and enzyme activity were consistently up-regulated in *K-ras*–transformed pancreatic cancer cells. Previous studies have indicated that the triggers of NOX regulatory subunits involved protein kinases, lipid-metabolizing enzymes, and guanine-nucleotide exchange proteins that activate Rac [10]. It has also been known that phosphatidylinositol (3,4,5)-trisphosphate (PIP3), the lipid product of PI3K, activates Rac by binding to the pleckstrin homology (PH) domain of GEF, which mediates the exchange of Rac-GDP for Rac-GTP [27,28]. Because PI3K is one major downstream effector of K-ras, we postulated that K-ras induces the constitutive activation of NOX through the PI3K/PIP3/GEF/Rac pathway (Figure 6). Accordingly, we found that the levels of both phosphorylated p40phox and active GTP-binding Rac were significantly up-regulated in *K-ras*–transformed E6E7 cells. Interestingly, the binding of PtdIns(3)P, a product of PI3K, to the phox homology (PX) domain of p40phox was previously reported to lead to the stimulation of ROS formation [29], indicating that PI3K could also mediate the Ras-induced activation of NOX through p40phox.

Therapeutic selectivity, or the preferential killing of cancer cells without significant toxicity to normal cells, is an important consideration in cancer chemotherapy. In the current study, the constitutive activation of NOX was observed in *K-ras*–transformed pancreatic cancer cells. The deregulation and overexpression of the NOX family members have been reported in a variety of malignancies, and certain subunits seem to be associated with *K-ras* transformation [4,13,30]. Interestingly, we recently reported an up-regulation of p22phox in clinical pancreatic cancer specimens [31], and Hiraga *et al*. [32] reported that NOX4-derived ROS signaling contributes to TGF-β–induced epithelial-mesenchymal transition (EMT) in pancreatic cancer cells. Therefore, NAD(P)H oxidase may be an attractive target for preferentially killing malignant cells and is likely to have broad therapeutic implications. Capsaicin, a natural compound found in peppers, has been found to interact with NOX and induce ROS generation [15]. Our study revealed that capsaicin caused selective cytotoxicity to *K-ras*–transformed cancer cells but minimum effect on parental E6E7 cells. Capsaicin was also able to inhibit the proliferation of primary pancreatic cancer AsPC-1 cells. In addition, capsaicin was able to inhibit invasion, the deadly feature of pancreatic cancer, in both *K-ras*–transformed pancreatic cancer cells and primary pancreatic cancer cell lines, Capan-1, Panc-1, and AsPC-1. Importantly, capsaicin induced a tremendous accumulation of ROS in *K-ras*–transformed cells compared with parental E6E7 cells. The *in vitro* assay of NOX activity showed that capsaicin preferentially inhibited NOX activity through the suppression of Rac, a NOX component, in *K-ras*–transformed cells, whereas only having minimal effects on parental E6E7 cells. DPI, another NOX inhibitor, also exhibited a selective depletion of ATP in *K-ras*–transformed pancreatic cancer cells as well as primary pancreatic cancer cells. Furthermore, our animal study showed that capsaicin has therapeutic activity in mice bearing pancreatic cancer xenografts. Examination of the tumor sections demonstrated the oxidative damage induced by capsaicin and suggesting the role of ROS accumulation in capsaicin-induced cell death.

Compared with normal cells, cancer cells are under sustained oxidative stress due to the presence of constant oncogenic signals and hence are highly dependent on antioxidants to counterbalance ROS stress [33]. Therefore, further oxidative insults, such as the exposure to ROS-generating agents, could exhaust the cellular antioxidant capacity and cause severe accumulation of ROS, leading to cell death. In contrast, it is less likely to induce such severe ROS stress in normal cells, due to their low basal ROS levels. This biochemical difference between normal and cancer cells may constitute a basis for modulating cellular ROS as a strategy to selectively kill cancer cells. Our study demonstrated the significant increase in ROS in *K-ras*–transformed cells compared with parental E6E7 cells. Importantly, capsaicin induced further ROS accumulation in *K-ras*–transformed cells, but had little effects on parental E6E7 cells. Consistently, capsaicin significantly inhibited the growth and invasion of *K-ras*–transformed cells, whereas the identical concentration did not show any toxicity on parental E6E7 cells.

## Conclusions

In conclusion, our results demonstrated the up-regulation and activation of NOX, the ROS-generating enzyme, by *K-ras* transfection. Our study suggests that the intrinsic oxidative stress associated with *K-ras*–induced oncogenic transformation provides a basis for developing strategies to specifically target pancreatic cancer cells through a redox-mediated mechanism. Considering the frequent mutation of *K*-*ras* in pancreatic cancer and its resistance to many anticancer agents, targeting NOX may have significant clinical implications and warrant further investigation.
